# Quality of Life, Insomnia and Coping Strategies during COVID-19 Pandemic in Hospital Workers. A Cross-Sectional Study

**DOI:** 10.3390/ijerph182312466

**Published:** 2021-11-26

**Authors:** Sebastiano Italia, Chiara Costa, Giusi Briguglio, Carmela Mento, Maria Rosaria Anna Muscatello, Angela Alibrandi, Francesca Larese Filon, Giovanna Spatari, Michele Teodoro, Concettina Fenga

**Affiliations:** 1 Department of Biomedical and Dental Sciences and Morphofunctional Imaging, Occupational Medicine Section, University of Messina, 98125 Messina, Italy; sebastos87@hotmail.it (S.I.); giusi.briguglio@hotmail.it (G.B.); gspatari@unime.it (G.S.); cfenga@unime.it (C.F.); 2 Clinical and Experimental Medicine Department, University of Messina, 98125 Messina, Italy; ccosta@unime.it; 3 Department of Biomedical and Dental Sciences and Morphofunctional Imaging, Clinical Psychology, Psychiatric Unit, University of Messina, 98125 Messina, Italy; carmela.mento@unime.it; 4 Department of Biomedical and Dental Sciences and Morphofunctional Imaging, Psychiatric Unit, University of Messina, 98125 Messina, Italy; maria.muscatello@unime.it; 5 Department of Economics, University of Messina, 98125 Messina, Italy; angela.alibrandi@unime.it; 6 Department of Medical Sciences, Clinical Unit of Occupational Medicine, University of Trieste, 34100 Trieste, Italy; larese@units.it

**Keywords:** COVID-19, hospital workers, quality of life, coping strategy, insomnia

## Abstract

COVID-19 became a pandemic in a few months, leading to adverse health outcomes, reducing the quality of life, affecting the sleep/wake cycle, and altering coping strategies, especially among hospital personnel. Life quality, insomnia, and coping strategies were thus assessed among hospital personnel during the first wave of the COVID-19 pandemic in Italy. This cross-sectional study was conducted from May to November 2020 through an online survey. There were 558 participants (28.5% males and 71.5% females) enrolled in two different metropolitan areas (in North and South of Italy, respectively). Three standardized questionnaires were administered: European Quality of life–5 Dimensions (EQ-5D), Athens Insomnia Scale (AIS), and Brief COPE. Differences in sociodemographic characteristics and work-related factors were also investigated in order to identify possible predictors through a generalized linear model and logistic regression analysis. Results showed good perceived life quality and high insomnia prevalence. After sample stratification, the statistical analysis highlighted that personal (gender, age, educational level) and work-related factors (employment in COVID wards, remote working) played different roles in predicting quality of life, insomnia, and coping attitude. Active, Planning, and Acceptance were the most frequently adopted coping strategies. Despite women confirming their attitude in reacting to the difficulties, adopting emotion-focused coping strategies, they showed a higher probability to develop insomnia, so a gender perspective should be considered in the health protection of this working category. An integrated approach should be implemented at individual, interpersonal and organizational levels aiming to monitor psychological distress, favor regular sharing and communication between peers, and also allow conciliation of work with family life. At the organizational level, preventive and protective measures adequate to work-related risk to COVID-19 should be adopted.

## 1. Introduction

Coronavirus disease (COVID-19), which started in Wuhan, China, in December 2019, became a pandemic in a few months, leading to extraordinary risks to human beings [1]. Despite the majority of infected subjects having a moderate illness and about 10–15% of patients developing grave complications [2], until 21 October 2021, about 4.9 million deaths were declared, with over 241 million cases confirmed globally [3]. 

In Italy, the epidemiological situation during the first wave, since February 2020, differently concerned the country with a significant burden of disease in the North rather than the South; in particular, Lombardy, Piedmont, Emilia Romagna, and Veneto were the most affected northern regions [4]. The Italian government handled this critical situation by implementing preventive measures and adopting a national lockdown on 10 March 2020 [5]. Consequently, Italians lived in social isolation for about two months; only indispensable activities were allowed and leaving home was consented to only for health reasons, purchasing vital products, and reaching the workplace, when permitted [6]. The pandemic altered everybody’s lives and work behaviors, particularly those healthcare workers (HCWs) who were involved on the frontline with increased exposure to SARS-CoV-2 infection, lack of validated guidelines, and shortage of resources including personal protective equipment [7]. In addition, these workers have often decided to live far from their loved ones to keep them safe from an additional risk of contagion [8].

In previous research, outbreaks of other contagious diseases led to adverse health outcomes in HCWs impacting physical, social, emotional, or spiritual wellbeing, globally reducing the quality of life [9,10,11]. Despite life quality being a broad-range concept, the WHO defines it as the subjective perception of own position in life in the specific cultural context and in relation to personal expectations, standards, and concerns [12]. The literature describes five dimensions that define life quality in terms of mobility, self-care, usual activities, pain/discomfort, and anxiety/depression [13]. The current COVID-19 pandemic has created circumstances with overwhelming stressors on HCWs, through increased working loads, high risk of exposure to SARS-CoV-2, and overall disruptions of daily life, leading to increased anxiety, stress, depression, burnout and sleep disorders [14], especially insomnia [15], and to a drastic reduction in the perceived quality of life [16,17]. 

The considerable psychological impact of the COVID-19 pandemic has undoubtedly influenced feelings and behaviors [18,19], requiring the adoption of coping strategies to play a buffering role on stress and have a preventive effect on mental health [20]. Different coping strategies are used depending on external factors (such as cultural and workplace context or geographical area) [21] and individual components (e.g., rage, terror, or sadness) [22].

Though it has been demonstrated that the trend of contagion has differently affected the mental health status of HCWs working in areas with dissimilar incidences of COVID-19 cases [23,24], it is also true that regional differences in stress perception and coping strategies also depend on cultural factors, home/work interface, social support, and economic environment [25,26]. In a Chinese study, comparing subjects coming from Hubei and from non-endemic provinces, health workers in the endemic region showed lower anxiety levels about the COVID-19 epidemic [23]. In a multicentre prospective cohort epidemiological study, the regional origin explained a small fraction of differences in perceived job stress [27], while other factors seem to play major roles in affecting this aspect. For example, family is a fundamental source of support, particularly in developing areas where social services are scarce [28]. Under these premises, we mainly aimed to assess the quality of life, insomnia, and analyze the different coping strategies adopted among hospital personnel during the first wave of the COVID-19 pandemic in Italy. More specifically, we examined the differences in sociodemographic characteristics and work-related factors in two different Italian metropolitan areas with similar epidemiological trends, located in the North and in the South of Italy, respectively. We intended to identify eventual work-related and sociodemographic predictors of worse outcomes, suggesting insights on the best tailored preventive and organizational measures in the workplace. 

## 2. Materials and Methods

### 2.1. Study Design and Population

This cross-sectional study was conducted from May to November 2020 through an online survey. Participants were enrolled among hospital personnel working in different medical treatment facilities and included physicians, nurses, and other employees (such as biologists, pharmacists, laboratory technicians, and office workers). According to Italian legislation, in order to reduce the number of SARS-CoV-2 infections in the workplaces, employers had the possibility, when applicable, to guarantee working from home for the most vulnerable subjects. Consequently, some office workers enrolled in the present investigation performed remote work.

Study subjects were recruited in two Italian metropolitan areas, namely Trieste (group N) in the North and Messina (group S) in the South of Italy. 

Data were collected through an online platform recruiting subjects by spreading an invitation link. In order to increase the diffusion and validity of this sampling method, the invitation for the survey was sent to directors and coordinators, requesting them to spread it to their teams in a hierarchical line.

### 2.2. Procedures and Measures

The self-administered questionnaire was composed of two sections and took no more than twenty minutes to be completed. The first section investigated the sample’s sociodemographic characteristics and work-related factors: gender, age, educational degree, marital status, number of children, profession, employment in COVID wards, number of contacts per week with COVID patients, remote working, and seniority. The second one comprised three standardized questionnaires: European Quality of life–5 Dimensions (EQ-5D), Athens Insomnia Scale (AIS), and Brief COPE.

The European Quality of life–5 Dimensions (EQ-5D) is a broadly used questionnaire developed in Europe to evaluate the essential quality of life components. This tool measures mobility, self-care, usual activities, pain/discomfort, and anxiety/depression through one question for each of the five dimensions. Throughout an algorithm, the given answers permit the calculation of the EQ-5D index, in which 0 is death and 1 represents perfect health. The EQ-5D questionnaire also comprises a Visual Analog Scale (VAS), measuring respondents’ perceived health status, ranging from 0 (the worst thinkable wellbeing) to 100 (the best thinkable wellbeing) [29]. Specifically, the EQ-5D index value describes the health state, while the EQ-VAS gives information about individual health perception [30,31].

The Athens Insomnia Scale (AIS) is an eight-item questionnaire that reveals insomnia. The first five questions report the subject’s nocturnal symptoms, while the last three items investigate the daytime impact due to sleep disorders. Each item is assigned a score from 0 to 3 according to a 4-point Likert scale (with 0 equivalent to “no problem” and 3 to a “severe problem”). The maximum total score is 24, which indicates the most severe insomnia symptoms. A cut-off of ≥6 represents the criterion for confirming insomnia symptoms [32].

The Brief COPE evaluates different coping strategies, both adaptation and maladaptation approaches. We used this tool to evaluate the stress response in a recent period (“situational-actual” version). The questionnaire includes 28 items, each assigned a score from 1 to 4 according to a 4-point Likert scale, divided into 14 factors, each consisting of two items. The 14 factors are Self-Distraction; Active Coping; Denial; Substance Use; Emotional Support; Instrumental Support; Behavioral Disengagement; Venting; Positive Reframing; Planning; Humor; Acceptance; Religion and Self-Blame [33]. 

### 2.3. Ethical Issues

This study was carried out in accordance with the Declaration of Helsinki’s ethical standards. The study needed no formal approval by the local Ethics Committee, though a formal communication of study beginning was given (notification with request for acknowledgement). All the subjects who accepted voluntary participation in the survey provided informed consent. Participation was voluntary and without compensation. 

### 2.4. Statistical Analysis

Descriptive analyses were performed for all variables; in particular, categorical variables were expressed as frequency and proportion, whilst continuous variables were expressed as mean and standard deviation. To determine differences between groups in categorical variables, we used chi-square tests and Fisher’s exact tests, as appropriate. After applying the Kolmogorov-Smirnov test and verifying the non-Gaussian distribution in most continuous variables, the differences between groups were evaluated using the Mann-–Whitney U test. The reliability of the three standardized questionnaires was evaluated by assessing their internal consistency through the computation of Chronbach’s alpha. Furthermore, in order to identify possible predictors of outcomes considered in the current investigation, we adopted different models: we used the generalized linear models for EQ-5D-Index, for EQ-VAS, and for each one of the 14 coping strategies of Brief-COPE; in addition, we estimated univariate and multivariate logistic regression models for Athens Insomnia Scale (dichotomized variable in according to previously described criterion). *p* values < 0.05 were considered statistically significant and reported in bold characters in the Tables. Statistical analysis was performed using IBM SPSS Statistics 23 (IBM Corp, Armonk, NY, USA).

## 3. Results

A total of 558 respondents, 347 participants in group N and 211 in group S, accepted to participate in the study and completed the survey. A detailed description of the study population is summarized in Table 1. 

The study population consisted of 399 women (71.5%) and 159 men (28.5%) aged 18–65 years. We found statistically significant differences between the two groups in all the considered sociodemographic characteristics: the number of women in group N was higher than in group S (75.2% and 65.4%, respectively); less than one-third of subjects in group N (27.4%) and the majority in group S (56.9%) were aged under 40 years; most participants in group S were graduated (55%), while in group N the percentages were more equally distributed among the different educational degree. Regarding marital status, in group S, single (not married and divorced) and in pairs (married and unmarried partners) were similarly represented, whilst in group N, the majority had a partner (72.6%) and parenthood was more frequent in group N than in group S (59.7% and 45.5% had children, respectively).

Considering work-related factors, most of the participants were nurses in group N and doctors in group S; in both groups, there were no statistical differences in relation to the employment in COVID wards and the number of contacts per week with COVID patients. Moreover, 68 subjects (42 in group S and 26 in group N) were employed in remote working during the pandemic. In addition, we observed a higher length of employment in group N than in group S, with a statistically significant difference.

European Quality of life–5 Dimensions (Index and VAS), Athens Insomnia Scale and Brief COPE scores are reported in Table 2. The reliability assessment showed the following Chronbach’s alpha: EQ–5 D Index 0.59; Athens Insomnia Scale 0.86; while for the different coping strategies we found Active 0.70; Planning 0.74; Positive Reframing 0.70; Acceptance 0.54; Humor 0.65; Religion 0.88; Emotional Support 0.81; Instrumental Support 0.79; Self Distraction 0.50; Denial 0.55; Venting 0.58; Substance Use 0.89; Disengagement 0.50; Self Blame 0.42.

Despite the two groups showing high values of self-reported quality of life, group S showed better scores than group N both in Index and VAS of EQ-5D questionnaire with statistically significant differences. Moreover, we stratified the sample into different subgroups according to sociodemographic and work-related variables, comparing the two groups. Subsequently, we found the highest values of EQ-5D-Index in the stratified group S, with statistically significant differences among women, graduated subjects, participants with no children, workers not employed in COVID wards. Moreover, a similar trend was observed in EQ-VAS, except for gender, for which statistical significance was found among men but not among women. Furthermore, in order to identify possible predictors of better scores, we used a generalized linear model for EQ-5D-Index as reported in Table 3. 

In the total sample, male gender, high education levels, and lower seniority were positive predictors of a better perceived quality of life according to EQ-5D-Index. Having a partner and lower seniority were considered predictors of a better quality of life respectively in group N and group S. For EQ-VAS (Table 4), male gender and high education levels in the total sample represented significant predictors of better perceived quality of life. High education degree was identified as a positive predictor both in group N and S; while in group S male gender and lower seniority were considered predictors of more excellent scores in the European Quality of life questionnaire.

Differently, the Athens Insomnia Scale questionnaire revealed insomnia in 162 out of 247 subjects (46.7% in group N) and 91 out of 211 (43.1% in group S), without statistically significant differences. Nevertheless, after stratifying the sample as described above, we found statistically significant differences among not married subjects and participants with no children, showing worse outcomes in group N after stratification. Moreover, in the distribution of the Athens Insomnia Scale, we considered the score 6 as pathological cut-off (such as proposed by Soldatos et al. [32]); consequently, we used univariate and multivariate logistic regression (Table 5) in order to individuate significant predictors of insomnia symptoms. 

Accordingly with univariate logistic regression, female subjects (OR 2.09, 95% CI 1.42–3.07) and nurses (OR 1.62, 95% CI 1.09–2.42), both male and female, showed a high risk of suffering from insomnia in the total sample, while multivariate approach showed only women as the category at high risk (OR 2.20, 95% CI 1.48–3.28), in the overall sample as well as in both groups N and S. In group N, single subjects (not married and divorced) showed a higher risk of suffering from insomnia (OR 1.76, 95% CI 1.09–2.83) in univariate regression. In group S univariate approach showed that the number of contacts per week with COVID patients was also a work-related factor determining a high risk of insomnia (OR 1.29, 95% CI 1.00–1.66); moreover, in the multivariate logistic regression, nurses showed a lower risk of insomnia when compared to physicians (OR 0.99, 95% CI 0.98–0.99).

Considering the mean scores of the Brief COPE questionnaire (Table 2), the coping strategies with the highest values were Active, Planning and Acceptance, while Substance Use and Disengagement reported the lowest scores in both groups. Moreover, group S reported higher values than group N in Humor, Religion, Denial, and Self-blame, showing statistically significant differences. Additionally, we applied a generalized linear model for each one of the 14 coping strategies. In the overall sample, we found different predictive variables as illustrated in Table 6A,B, for sociodemographic and work-related features of the study population, respectively. Male gender was revealed to be the most frequently described negative predictor in our statistical models, showing that being a woman is related to almost all the analyzed coping strategies. An age of >40 y acted as a predictor of Acceptance and Religion; education positively predicted Emotional Support, while a lower educational level was in relation with Denial and Venting. Being part of group S predicted Religion and Denial, while group N participants were related to Instrumental Support. As regards work-related factors, the employment in COVID wards was related to Emotional and Instrumental Support. On the other hand, remote working predicted Religion, Denial, and Disengagement. No predictive variables were found for the coping strategies Positive reframing, Humor, and Substance use. While Disengagement was not predicted from any sociodemographic characteristics, no work-related variables were found as predictors of Acceptance, Self-distraction, Venting, and Self-blame.

## 4. Discussion

This study investigated the quality of life and insomnia among hospital personnel during the first wave of the COVID-19 pandemic in Italy. The adoption of different coping strategies was also analyzed. In particular, we investigated the differences in sociodemographic characteristics and work-related factors in two different Italian metropolitan areas, located in Northern and Southern Italy (group N and group S, respectively). We also identified work-related and sociodemographic predictors of specific outcomes.

Our results showed an overall good perceived quality of life despite a high prevalence of insomnia among the participants in both groups. The Brief-COPE questionnaire revealed that the subjects experienced adequate adaptive mechanisms, demonstrating that Active, Planning, and Acceptance were the most frequently adopted coping strategies in both groups.

The EQ-5D and EQ-VAS questionnaires showed good health status and perceived quality of life in both groups. We can hypothesize that this finding might be explained by different possible factors: low incidence of COVID-19 cases in the two metropolitan areas may have been adequately managed. Furthermore, since the survey was conducted during the first wave, the interviewed subjects may have underestimated the magnitude of the pandemic; another explanation might be found in a good level of organizational support with adequate provision of medical equipment and PPE (personal protective equipment). In particular, group S participants reported higher scores which their sociodemographic characteristics may explain: the majority of subjects was <40 y (56.9% vs. 27.4% in group N), the percentage of male participants was higher than group N (34.6% vs. 24.8%, respectively) and most of the interviewees were graduated (55% vs. 37.8% in group N). In fact, aging is associated with an increased burden of disease, and a higher education level is reported to confer knowledge and consciousness regarding the risk of infection and correct preventive measures, particularly in the COVID-19 pandemic [34,35,36,37,38].

Moreover, regarding work-related features, it can be highlighted that only in group S did high seniority act as a predictor of worse overall life quality, whereas working in COVID wards predicted its perception. This relation was not present in group N: probably, the organization of the healthcare system with a higher readiness level in the working context of this group may have played a role in buffering the negative impact of the pandemic on mental health and social life on HCWs [39,40]. In fact, the investigated northern metropolitan area was in proximity to the most affected Italian regions during the first pandemic wave.

As demonstrated in other research, in frontline hospital workers, working conditions increased the perception of personal threat, increasing stress levels with an inevitable worsening of the perception of health status and quality of life [41,42]. In contrast, another study on nurses reported that the social domain of quality of life had a significant positive association with working experience [43]. 

In our total sample, we found that high education level was a predictor of better perceived health status in the two study groups, in accordance with the existing literature [34,35,36]. In fact, as mentioned above, an elevated level of education generally corresponds to higher career profiles with greater earnings and a better perception of life quality as well as more robust mechanisms to face situations of initial disability or deterioration in health status. Moreover, male gender was related to better life quality, both overall (*p* < 0.001) and perceived (*p* < 0.05), confirming that men are more likely to report good scores when compared with women [37]; during this period of a whole disruption concerning many organizational aspects in daily life, the social pressure exerted by family may have negatively impacted the quality of life, especially in women. 

As is well known, the new living arrangement, mainly due to social distancing, has led to unprecedented social experiences, resulting in an increase of anxiety, stress, depression, burnout, and sleep disorders [14]. In particular, insomnia was revealed to be one of the most frequent disturbances [15]. In accordance with other research [44,45] and a recent meta-analysis [46], we found a high prevalence of insomnia in our study population, with almost half of participants reporting insomnia symptoms in both groups. Our data revealed that different factors in the two groups could represent a risk to the onset of insomnia. In group S, subjects with a higher number of contacts per week with COVID patients had a greater risk of insomnia. Literature suggests that working conditions linked to an elevated number of contacts with COVID-19 patients may justify the higher levels of distress, resulting in sleep problems [47,48,49]. 

The stratification of the study population by gender and professional category highlighted an increased risk of insomnia among women (OR 2.09, *p* < 0.001) and nursing personnel (OR 1.62, *p =* 0.018), similarly to other studies [48,49]. Evidence suggests that women are more susceptible to sleep disorders, also due to a double burden of work hanging on them [50]. Since women are more disposed to suffer from psychological symptoms, including mood disorders [51,52], subsequently to stressful events, the COVID-19 pandemic represented a traumatic component that may have revealed this greater vulnerability. These conditions may negatively influence sleep quality [53]. Though explaining this gender difference is not straightforward, individual features (e.g., genetics, hormones) and social disparities might represent the possible causes [54]. Additionally, the literature suggests that nurses are more exposed to the pandemic burden [49]. 

The female gender was also a predictor of higher scores in almost all coping strategies encountered by the Brief-COPE questionnaire, especially those related to support.

In general, women showed a more intense effort in their attempt to cope with the difficulties linked to the pandemic situation and were confirmed to be more likely to use emotion-focused coping strategies, while men tend to rely more on problem-focused strategies [55].

Concerning the capacity to handle stressful situations, the most commonly used strategies, equally adopted in both study groups, were those with a positive attitude towards the workplace (Active, Planning, and Acceptance), similar to previous studies on HCWs [56,57]. The functional coping strategies permit to favorably decode adverse circumstances, positively affecting mental wellbeing and life quality [58]. Following the application of the statistical model, in group N we only found a sociodemographic characteristic, age > 40 y, as a predictor of Acceptance; in fact, age could be considered as a protective characteristic against the development of stress and a greater individual experience may orientate coping to the adoption of positive strategies in this working population [59]. Differently, in group S data showed that a work-related factor, the number of contacts per week with COVID-19 patients, played a role in predicting Planning attitude. Contrary to other research in which greater exposure to SARS-CoV-2 infection has led HCWs to adopt maladaptive behaviors [58], this work-related factor in our Southern population acted as a positive stimulus in adopting a more functional coping strategy. We can hypothesize that there are not only demographic features but also cultural and environmental factors that can influence the use of this strategy, so a higher workload with challenging tasks seems to correspond to more significant planning activity. 

Moreover, the national lockdown and government restrictive preventive measures limited social relationships also outside the work environment, with a consequent impact on coping strategies involving social support (emotional and instrumental support). Notwithstanding, our study population demonstrated to rely on social interactions, confirming other data in the literature [60,61]. In particular, being part of group N acted as a predictor of the Instrumental Support strategy, which is a problem-focused strategy whereby subjects seek information, advice, and assistance [62]. Considering the higher prevalence of the pandemic in most regions of Northern Italy, these subjects may have been more afraid to infect their families, leading them to the choice to live far from their loved ones [7], resulting in a greater search for social support, especially counseling and enlightenment.

Furthermore, our results showed a significant difference between the two groups: religion was a frequent mechanism in group S, particularly in older subjects and those working remotely; whereas in group N females and more COVID-exposed participants tended to practice their spirituality in critical situations [63]. Some people have shown a significant attitude to draw resources from their religious feelings in the current pandemic [64], although explaining individual motivations is not straightforward.

Working from home has resulted in being predictive of relying not only on religion but also on maladaptive coping strategies, particularly in group S of this population (Table 6B). The strategies aiming to avoidant behaviors (Self-distraction, Denial, and Disengagement) constitute a risk factor for elevated distress levels, in fact, they are categorized among dis-functional reactions to stressful situations [65,66]. Despite our investigation showing low scores in most of these strategies, group S was related to Denial, pretending that the situation was not real [67]. It is possible that due to cultural and environmental characteristics, these subjects tended to minimize the threat, keep feelings to themselves and avoid mental distress by making an effort to forget. 

Overall, our data underline that dissimilar variables play distinct roles in affecting coping tactics in the two geographical areas. Actually, as predictors for psychological distress depend on the specific context, also the consequent coping strategies are not absolute and depend on a multiplicity of variables.

The first limitation of this study is the cross-sectional design that does not permit to define the direction of causality. Second, despite the fact that we used all validated questionnaires, the online administration of a survey could be affected by a responder bias: the sample was recruited through network invitation, so enrolled subjects had to be able to use web resources. Finally, due to the self-administration of questionnaires, we cannot generalize our findings because of the risk of overestimating psychological disturbs and insomnia.

In spite of these limitations, the strength of this survey has been to evaluate the quality of life, insomnia, and coping strategies in facing COVID-19 physical and emotional burden, through the comparison of two groups residing in distinct Italian metropolitan areas with matching low SARS-CoV-2 incidence rate but dissimilar sociodemographic features and work-related factors. Only a few Italian investigations were conducted among different regions, assessing the impact of COVID-19 on HCWs, in terms of psychological safety and workload [57,68,69,70,71]. This kind of comparison has permitted us to achieve new insights on how sociodemographic characteristics and work-related factors may have played different roles depending on different organizational settings, in a preventive perspective.

Since the first year of this ongoing pandemic, the lesson learned is that, for a future similar emergency, public health authorities should implement support programs dedicated explicitly to more vulnerable personnel between HCWs. Given the gender-linked mental health challenges and coping attitudes, women would particularly benefit from psychosocial support delivered according to their work schedules to avoid interference with parental tasks. 

A multilevel integrated approach should be implemented on the individual HCW aiming to monitor psychological distress and help in accepting negative emotions; at the interpersonal dimension, to favor regular sharing and communication between peers, also to allow conciliation of work with family life; in particular, for remote workers, the organization of frequent online meetings could help in maintaining contact between co-workers and avoid disengagement. Moreover, at the organizational level, preventive and protective measures adequate to work-related risk to COVID-19 [72] should be adopted, allowing timely availability of clear information, guidelines, and protective equipment.

## 5. Conclusions

Globally, our study population reported good perceived quality of life and self-reported health status, despite the pandemic situation.

Women confirmed their attitude to positively react to the difficulties linked to the pandemic, adopting emotion-focused and support-related coping strategies. 

A high prevalence of insomnia was reported, particularly by women and nurses. Considering the high feminization of healthcare professions in western countries, as well as the higher probability for women to develop mental health disturbs, gender perspective should be considered at the organizational level; we suggest enhancing health protection actions dedicated to these more vulnerable categories, through prevention and intervention programs oriented towards psychosocial support to mitigate the impact of stressful events, such as the COVID-19 pandemic.

## Figures and Tables

**Table 1:** Description of study population: sociodemographic characteristics and work-related factors.

		Total	Group N	Group S	
		*n* (%)	*n* (%)	*n* (%)	*p*-Value
SOCIODEMOGRAPHIC FACTORS					
Total		558 (100)	347 (62.2)	211 (37.8)	
Gender					
	Male	159 (28.5)	86 (24.8)	73 (34.6)	**0.013**
	Female	399 (71.5)	261 (75.2)	138 (65.4)	
Age					
	<40 y	215 (38.5)	95 (27.4)	120 (56.9)	**<0.001**
	>40 y	343 (61.5)	252 (72.6)	91 (43.1)	
Education					
	Middle school	14 (2.5)	13 (3.7)	1 (0.2)	**<0.001**
	High School	108 (19.4)	83 (23.9)	25 (11.8)	
	Graduation	247 (44.3)	131 (37.8)	116 (55.0)	
	Post-graduation	189 (33.9)	120 (34.6)	69 (32.7)	
Marital status					
	Not married	135 (24.2)	62 (17.9)	73 (34.6)	**<0.001**
	Unmarried partners	117 (21.0)	86 (24.8)	31 (14.7)	
	Married	258 (46.2)	166 (47.8)	92 (43.6)	
	Divorced	48 (8.6)	33 (9.5)	15 (7.1)	
Parenthood					
	No	255 (45.7)	140 (40.3)	115 (54.5)	**0.001**
	Yes	303 (54.3)	207 (59.7)	96 (45.5)	
Number of children					
	Mean ± SD	0.96 ± 1.06	1.04 ± 1.03	0.82 ± 1.11	**0.003**
WORK-RELATED FACTORS					
Profession					
	Physician	184 (33.0)	67 (19.3)	117 (55.5)	**<0.001**
	Nurse	212 (38.0)	154 (44.4)	58 (27.5)	
	Others	162 (29.0)	126 (36.3)	36 (17.1)	
COVID Ward					
	No	450 (80.6)	282 (81.3)	168 (79.6)	0.633
	Yes	108 (19.4)	65 (18.7)	43 (20.4)	
Number of contacts per week with COVID patients					
	None	269 (48.2)	160 (46.1)	109 (51.7)	0.471
	One	81 (14.5)	49 (14.1)	32 (15.2)	
	Five	139 (24.9)	93 (26.8)	46 (21.8)	
	Exclusive	69 (12.4)	45 (13.0)	24 (11.4)	
Remote working					
	No	490 (87.8)	321 (92.5)	169 (80.1)	**<0.001**
	Yes	68 (12.2)	26 (7.5)	42 (19.9)	
Seniority (years)					
	Mean ± SD	16.17 ± 12.62	18.97 ± 12.75	11.56 ± 10.96	**<0.001**

**Table 2:** Mean scores of validated questionnaires assessing health-related and perceived quality of life, insomnia, and coping strategies in healthcare personnel during the first wave of COVID-19 pandemic (*n* = 558).

		Total		Group N		Group S		
		Mean ± SD		Mean ± SD		Mean ± SD		*p*-Value
EQ–5D–Index		0.785 ± 0.230		0.764 ± 0.226		0.821 ± 0.232		**<0.001**
EQ–VAS		75.70 ± 17.51		74.50 ± 17.07		77.68 ± 18.18		**0.004**
Athens Insomnia Scale								
	Mean ± SD	5.76 ± 3.96		5.87 ± 3.92		5.57 ± 4.02		0.252
	≥6 (%)	253 (45.3)		162 (46.7)		91 (43.1)		0.413
Brief–COPE								
	Active	6.53 ± 1.37		6.57 ± 1.29		6.47 ± 1.51		0.877
	Planning	6.56 ± 1.32		6.57 ± 1.24		6.55 ± 1.45		0.578
	Positive Reframing	5.51 ± 1.58		5.55 ± 1.55		5.43 ± 1.62		0.396
	Acceptance	6.11 ± 1.32		6.14 ± 1.22		6.05 ± 1.48		0.943
	Humor	3.72 ± 1.46		3.61 ± 1.40		3.91 ± 1.55		**0.029**
	Religion	3.66 ± 1.87		3.40 ± 1.81		4.09 ± 1.89		**<0.001**
	Emotional Support	4.49 ± 1.67		4.51 ± 1.64		4.47 ± 1.71		0.697
	Instrumental Support	4.91 ± 1.64		4.98 ± 1.53		4.78 ± 1.80		0.116
	Self Distraction	5.24 ± 1.59		5.22 ± 1.59		5.26 ± 1.60		0.913
	Denial	2.78 ± 1.19		2.63 ± 1.06		3.01 ± 1.34		**0.001**
	Venting	4.45 ± 1.50		4.53 ± 1.47		4.32 ± 1.55		0.111
	Substance Use	2.25 ± 0.83		2.22 ± 0.76		2.31 ± 0.94		0.426
	Disengagement	2.82 ± 1.15		2.80 ± 1.08		2.86 ± 1.26		0.993
	Self Blame	5.03 ± 1.44		4.89 ± 1.35		5.25 ± 1.56		**0.009**

**Table 3:** Generalized linear model for EQ-5D-Index, assessing quality of life in healthcare workers during the first wave of COVID-19 pandemic (*n* = 558).

Independent Variables		B-Value	95% CI	*p*-Value
Total				
	Sex (male)	0.08	0.04–0.12	**<0.001**
	Age (>40 y)	−0.02	−0.08–0.04	0.570
	Education	0.03	0.01–0.05	**0.029**
	Marital status (married)	0.03	−0.01–0.07	0.128
	Parenthood	−0.02	−0.07–0.02	0.294
	Region (south)	0.02	−0.02–0.06	0.429
	Profession (nurse)	0.01	−0.01–0.01	0.824
	COVID ward (yes)	−0.01	−0.06–0.05	0.841
	N° contacts with COVID patients per week	−0.01	−0.03–0.01	0.329
	Remote working (yes)	0.01	−0.05–0.07	0.732
	Seniority (years)	−0.01	−0.02–−0.01	**0.007**
Group N				
	Sex (male)	0.09	0.04–0.14	**0.001**
	Age (>40 y)	−0.03	−0.11–0.04	0.367
	Education	0.03	0.01–0.05	0.069
	Marital status (married)	0.06	0.01–0.11	**0.036**
	Parenthood	−0.01	−0.06–0.04	0.717
	Profession (nurse)	0.01	−0.01–0.01	0.668
	COVID ward (yes)	0.01	−0.07–0.06	0.911
	N° contacts with COVID patients per week	0.01	−0.02–0.03	0.975
	Remote working (yes)	−0.01	−0.10–0.08	0.782
	Seniority (years)	0.01	−0.01–0.01	0.238
Group S				
	Sex (male)	0.09	0.02–0.15	**0.007**
	Age (>40 y)	0.07	−0.03–0.18	0.166
	Education	0.04	−0.01–0.08	0.130
	Marital status (married)	−0.01	−0.08–0.05	0.686
	Parenthood	−0.02	−0.10–0.06	0.653
	Profession (nurse)	0.01	−0.01–0.01	0.558
	COVID ward (yes)	−0.02	−0.05–0.01	0.907
	N° contacts with COVID patients per week	−0.02	−0.05–0.01	0.126
	Remote working (yes)	0.01	−0.08–0.08	0.973
	Seniority (years)	−0.01	−0.02–−0.01	**<0.001**

**Table 4:** Generalized linear model for EQ-VAS, assessing perceived wellbeing in healthcare workers during the first wave of COVID-19 pandemic (*n* = 558).

Independent Variables		B-Value	95% CI	*p*-Value
Total				
	Sex (male)	3.36	0.16–6.55	**0.039**
	Age (>40 y)	−3.42	−8.06–1.21	0.148
	Education	2.59	0.74–4.44	**0.006**
	Marital status (married)	1.44	−1.84–4.72	0.390
	Parenthood	−1.84	−5.35–1.67	0.303
	Region (south)	−0.17	−3.35–3.02	0.919
	Profession (nurse)	0.01	−0.01–0.01	0.145
	COVID ward (yes)	−1.65	−5.69–2.40	0.425
	N° contacts with COVID patients per week	0.78	−0.73–2.28	0.312
	Remote working (yes)	2.49	−2.08–7.06	0.285
	Seniority (years)	−0.12	−0.29–0.05	0.180
Group N				
	Sex (male)	0.97	−3.21–5.15	0.649
	Age (>40 y)	−5.57	−11.36–0.23	0.060
	Education	2.46	0.27–4.64	**0.028**
	Marital status (married)	1.49	−2.73–5.71	0.488
	Parenthood	−0.03	−4.29–4.23	0.989
	Profession (nurse)	0.01	−0.01–0.01	0.232
	COVID ward (yes)	1.88	−.34–7.22	0.488
	N° contacts with COVID patients per week	0.17	−1.77–2.12	0.862
	Remote working (yes)	−0.35	−7.43–6.73	0.922
	Seniority (years)	0.02	−0.17–0.22	0.833
Group S				
	Sex (male)	7.50	2.52–12.48	**0.003**
	Age (>40 y)	4.95	−3.14–13.03	0.229
	Education	3.57	0.02–7.11	**0.048**
	Marital status (married)	1.90	−3.34–7.15	0.475
	Parenthood	−3.56	−9.76–2.65	0.260
	Profession (nurse)	0.01	0.00–0.01	0.478
	COVID ward (yes)	−6.93	−13.18–−0.67	**0.030**
	N° contacts with COVID patients per week	1.95	−0.43–4.33	0.108
	Remote working (yes)	2.00	−4.06–8.06	0.515
	Seniority (years)	−0.66	−1.03–−0.29	**<0.001**

**Table 5:** Univariate and multivariate logistic regression for Athens Insomnia Scale in healthcare workers during the first wave of COVID-19 pandemic (*n* = 558).

		UNIVARIATE			MULTIVARIATE		
Independent Variables		OR	95% CI	*p*-Value	OR	95% CI	*p*-Value
Total							
	Sex (female)	2.09	1.42–3.07	**<0.001**	2.20	1.48–3.28	**<0.001**
	Age (>40 y)	1.15	0.81–1.62	0.434	1.46	0.65–2.01	0.636
	Education	0.89	0.72–1.10	0.281	0.91	0.73–1.13	0.391
	Marital status (married)	0.82	0.58–1.17	0.275	0.81	0.55–1.21	0.304
	Parenthood	0.96	0.69–1.34	0.814	0.94	0.61–1.43	0.761
	Region (south)	0.87	0.61–1.22	0.413	0.99	0.68–1.46	0.975
	Profession (nurse)	1.62	1.09–2.42	**0.018**	1.00	1.00–1.01	0.674
	COVID ward (yes)	1.15	0.76–1.75	0.514	0.91	0.56–1.48	0.705
	N° contacts with COVID patients per week	1.30	0.93–1.81	0.127	1.20	1.00–1.44	0.057
	Remote working (yes)	0.72	0.43–1.21	0.211	0.77	0.44–1.35	0.771
	Seniority (years)	1.00	0.99–1.02	0.624	1.00	0.98–1.02	0.997
Group N							
	Sex (female)	2.19	1.31–3.65	**0.003**	2.27	1.34–3.85	**0.002**
	Age (>40 y)	1.08	0.67–1.74	0.744	1.36	0.67–2.78	0.393
	Education	0.89	0.70–1.14	0.371	0.91	0.70–1.18	0.470
	Marital status (married)	0.57	0.35–0.92	**0.021**	0.62	0.37–1.03	0.065
	Parenthood	0.76	0.50–1.17	0.216	0.77	0.46–1.30	0.324
	Profession (nurse)	1.00	1.00–1.01	0.247	1.00	1.00–1.01	0.128
	COVID ward (yes)	1.05	0.61–1.80	0.857	0.96	0.50–1.84	0.895
	N° contacts with COVID patients per week	1.05	0.87–1.27	0.612	1.14	0.90–1.45	0.281
	Remote working (yes)	0.83	0.37–1.85	0.642	0.78	0.33–1.85	0.569
	Seniority (years)	0.99	0.98–1.02	0.885	0.99	0.97–1.02	0.635
Group S							
	Sex (female)	1.93	1.07–3.48	**0.030**	2.81	1.46–5.38	**0.002**
	Age (>40 y)	1.15	0.66–1.99	0.623	0.76	0.27–2.16	0.607
	Education	0.91	0.60–1.38	0.652	0.98	0.63–1.54	0.932
	Marital status (married)	0.79	0.45–1.38	0.406	1.30	0.66–2.55	0.446
	Parenthood	1.32	0.77–2.29	0.316	1.22	0.55–2.72	0.626
	Profession (nurse)	0.99	0.99–1.00	0.099	0.99	0.98–0.99	**0.033**
	COVID ward (yes)	1.34	0.68–2.62	0.398	0.94	0.42–2.10	0.883
	N° contacts with COVID patients per week	1.29	1.00–1.66	**0.050**	1.34	0.99–1.83	0.058
	Remote working (yes)	0.68	0.34–1.37	0.280	0.96	0.44–2.10	0.914
	Seniority (years)	1.01	0.98–1.03	0.484	1.01	0.96–1.06	0.649

**Table 6:** (**A**). Generalized linear model for Brief-COPE in relation to sociodemographic predictors in healthcare workers (*n* = 558). (**B**). Generalized linear model for Brief-COPE in relation to work-related predictors in healthcare workers (*n* = 558).

(A)						
	Sociodemographic Characteristics					
Coping Strategies	Male	Age > 40 y	Education	Married	Parenthood	Southern Area
Active	−0.38 *^T^ (−0.68 to −0.07); −0.50 *^S^ (−0.97 to −0.04)					
Planning	−0.35 *^T^ (−0.64 to −0.05)					
Acceptance		0.64 *^N^ (0.12 to 1.15)				
Religion	−0.39 *^T^ (−0.78 to −0.01); −0.61 *^N^ (−1.15 to −0.07)	0.71 *^T^ (0.09 to 1.32) 1.57 **^S^ (0.52 to 2.63)				0.88 ***^T^ (0.47 to 1.29)
Emotional Support	−0.76 ***^T^ (−1.12 to −0.40); −0.98 ***^N^ (−1.47 to −0.49)		0.45 *^S^ (0.01 to 0.89)			
Instrumental Support	−0.67 ***^T^ (−1.03 to −0.32); −0.72 **^N^ (−1.18 to −0.26)					−0.44*^T^ (−0.81 to −0.06)
Self Distraction	−0.40 *^T^ (−0.73 to −0.06)				−0.48 *^T^ (−0.88 to −0.09)	
Denial	−0.39 *^N^ (−0.74 to −0.04)		−0.29 *^N^ (−0.55 to −0.03)			0.53 ***^T^ (0.25 to 0.81)
Venting	−0.58 ***^T^ (−0.91 to −0.25); −0.85 ***^N^ (−1.30 to −0.40)		−0.22 *^N^ (−0.42 to −0.01)			
Self Blame	−0.45 **^T^ (−0.75 to −0.15); −0.53 *^S^ (−0.97 to −0.09)					
**(B)**						
	Work-Related Factors					
Coping Strategies	Nurse	COVID Ward	COVID Patients	Remote Work		Seniority
Active	−0.41 *^T^ (−0.78 to −0.41)					
Planning			0.27 *^S^ (0.04 to 0.50)			
Religion			0.26 *^N^ (0.02 to 0.50)	0.81 *^T^ (0.17 to 1.45); 0.80 *^S^ (0.04 to 1.56)		
Emotional Support		0.61 **^T^ (0.16 to 1.06); 0.79 **^N^ (0.24 to 1.34)				−0.03 *^T^ (−0.05 to −0.01); −0.03 *^N^ (−0.05 to −0.01)
Instrumental Support		0.98 *^S^ (0.19 to 1.78)				
Denial	0.5 6 *^S^ (0.01 to 1.12)			0.47 *^T^ (0.02 to 0.91); 0.65 *^S^ (0.10 to 1.20)		0.04 *^S^ (0.01 to 0.07)
Disengagement				0.45 *^T^ (0.02 to 0.89); 0.64 *^S^ (0.10 to 1.18)		

Table reports B-values; 95% CI (in brackets); ^T^ = Total sample; ^N^ = Group N; ^S^ = Group S; * = *p*-value < 0.05; ** = *p*-value < 0.01; *** = *p*-value < 0.001. No predictive variables were found for the coping strategies Positive reframing, Humor, Substance use and Disengagement. Acceptance, Humor, Self-distraction, Venting, Substance use and Self-blame.

## Data Availability

The datasets generated and analyzed during the current study are available from the corresponding author on reasonable request.
